# Sniffer dogs as a screening/diagnostic tool for COVID-19: a proof of concept study

**DOI:** 10.1186/s12879-021-05939-6

**Published:** 2021-03-05

**Authors:** Esmaeil Eskandari, Milad Ahmadi Marzaleh, Hassan Roudgari, Ramin Hamidi Farahani, Amir Nezami-Asl, Reza Laripour, Helen Aliyazdi, Arasb Dabbagh Moghaddam, Ramin Zibaseresht, Hossein Akbarialiabad, Mojtaba Yousefi Zoshk, Hamidreza Shiri, Mahdi Shiri

**Affiliations:** 1 Researcher Relief and rescue Organization and SK9 Dogs Training School, Red crescent society of Islamic Republic of Iran, Tehran, Iran; 2 Research Center for Emergency and Disaster Resilience, Red Crescent Society of the Islamic Republic of Iran, Tehran, Iran; 3 Research Center for Health Management in Mass Gathering, Red Crescent society of the Islamic Republic of Iran, Tehran, Iran; 4 Department of Health in Disasters and Emergencies, Health Human Resources Research Center, School of Management and Medical Informatics, Shiraz University of Medical Sciences, Shiraz, Iran; 5 Health Policy Research Center, Institute of Health, Shiraz University of Medical Sciences, Fars, Iran; 6 Helal- Iran Institute, Red Crescent Society of the Islamic Republic of Iran, Tehran, Iran; 7 Genomics Research Centre, Shahid Beheshti University of Medical Sciences, Tehran, Iran; 8 Department of Research at Iran Medical Council, Tehran, Iran; 9 Head of Research Department at Iran Medical Council, Tehran, Iran; 10 Department of Infectious Diseases, Faculty of Medicine, AJA University of Medical Sciences, Tehran, Iran; 11 Medical Faculty of Aerospace and Subaquatic Medicine, AJA University of Medical Sciences, Tehran, Iran; 12 Researcher Center for Educational Research in Medical Sciences, Iran University of Medical Sciences, Tehran, Iran; 13 Researcher SK9 Dogs Training School, Shahriar, SK9 Dogs Training School, Tehran, Iran; 14 Researcher Department of Food Science, AJA University of Medical Sciences, Tehran, Iran; 15 Department of Chemistry (Christchurch) and Department of Chemistry and Physics, University of Canterbury and Maritime University of Imam Khomeini, Tehran, Iran; 16 Shiraz University of Medical Sciences, Shiraz, Iran; 17 Department of Pediatrics, AJA University of Medical Sciences, Tehran, Iran; 18 Islamic Azad University, Share-Qods Branch, Tehran, Iran; 19 Researcher Toxicology Research Center, AJA University of Medical Sciences, Tehran, Iran

**Keywords:** Diagnosis, Screen, Sniffer dogs, Covid-19, Health, Pharyngeal secretions

## Abstract

**Background:**

Sniffer dogs are able to detect certain chemical particles and are suggest to be capable of helping diagnose some medical conditions and complications, such as colorectal cancer, melanoma, bladder cancer, and even critical states such as hypoglycemia in diabetic patients. With the global spread of COVID-19 throughout the world and the need to have a real-time screening of the population, especially in crowded places, this study aimed to investigate the applicability of sniffer dogs to carry out such a task.

**Methods:**

Firstly, three male and female dogs from German shepherd (Saray), German black (Kuzhi) and Labrador (Marco) breeds had been intensively trained throughout the classical conditioning method for 7 weeks. They were introduced to human specimens obtained from the throat and pharyngeal secretions of participants who were already reported positive or negative for SARS-COV-2 infection be RT-PCR. Each dog underwent the conditioning process for almost 1000 times. In the meantime another similar condition process was conducted on clothes and masks of COVID-19 patient using another three male and female dogs from Labrador (Lexi), Border gypsy (Sami), and Golden retriever (Zhico) breeds. In verification test for the first three dogs, 80 pharyngeal secretion samples consisting of 26 positive and 54 negative samples from different medical centers who underwent RT-PCR test were in a single-blind method. In the second verification test for the other three dogs, masks and clothes of 50 RT-PCR positive and 70 RT-PCR negative cases from different medical center were used.

**Results:**

In verification test using pharyngeal secretion, the sniffer dogs’ detection capability was associated with a 65% of sensitivity and 89% of specificity and they amanged to identify 17 out of the 26 positive and 48 out of the 54 true negative samples. In the next verification test using patients’ face masks and clothes, 43 out of the 50 positive samples were correctly identified by the dogs. Moreover, out of the 70 negative samples, 65 samples were correctly found to be negative. The sensitivity of this test was as high as 86% and its specificity was 92.9%. In addition, the positive and negative predictive values were 89.6 and 90.3%, respectively.

**Conclusion:**

Dogs are capable of being trained to identify COVID-19 cases by sniffing their odour, so they can be used as a reliable tool in limited screening.

## Background

Since late 2019, the SARS-COV-2 virus has has started becoming a pandemic and to the current date, it affected millions of people and unfortunately killed hundreds of thousaunds so far [1] including medical staffs and involved all aspects of human’s life and businesses [2–4]. In the earlier phase of the pandemics, many countries used the quarantine policy to fight the spread of disease [5], however, the lockdowns caused a massive breakdown in economy, especially in developing and poor countries [6, 7]. The governments had to release the lockdowns at some points to save their economic, which has already resulted in the second wave of the pandemics [8].

The prime clinical modality in the diagnosis of COVID-19 seems to be Computed Tomography (CT-scan) and the golden standard method is the Polymerase Chain Reaction (PCR) test, that both of have limitations [9]. Studies showed that the PCR test for COVID-19 resulted in different sensitivity and specificity values across the world based on different factors including methods of obtaining samples [10–12]. Moreover, CT-scan requires radiation and raises the risk of multiple morbidities, including malignancies. Furthermore, both tests are neither suitable for public screening, nor are they available in all areas and settings [13, 14].

The meticulous canine olfactory system is well-known, which is equipped with an extensive scent epithelium (170 cm^2^ and 17 times greater than humans) as well as a large number of olfactory receptors (beyond 200 million receptors in comparison to 5 million in humans) [15, 16]. This ability is currently and commonly being used in detecting explosive materials, drugs, and dead bodies. The use of sniffer dogs in medical literature dates back to a case report in 1989, in which a dog could smell the mole of its owner [17]. When she wore a skirt, the dog wanted to bite one specific mole, while was indifferent about other moles on her body. The owner became suspicious and consulted a dermatologist, who came up with the diagnosis of melanoma. Since then many studies focused on the utility of dog olfaction for screening or diagnosis of different medical condition. The possible use of dogs in the diagnosis of some diseases such as malignancies, diabetes, Parkinson’s, seizures, and certain hormonal and enzymatic defects has been investigated by many studies [17–28]. It is commonly believed that dogs can be trained to detect the odour of particular molecules and compounds that changed during diseases. These odours are mainly believed to come from Volatile Organic Compounds (VOCs), produced by altered biochemical interactions inside body in the presence of malignancies, inflammations, infections, and other pathological events. Metabolic changes in the body result in a set of certain odours that may be recognisable for animals, specifically for dogs, as they have an extremly powerful olfactory system and also a very complex and unique analytical mechanism for interpretation of odours at their molecular levels [24]. Accordingly, COVID-19 should not be an exception. At the present time, the diagnosis of Covid-19 is based on sophisticated biochemical and genetic tests and CT-scan imaging. These modalities are relatively expensive and require accurate medical equipment, trained staffs and interpretation experts, which are not available everywhere across the world. Thus, the golden time may be lost for detecting affected people to cut the chain of transmission or to start the suitable treatments for affected people [29]. It is suggested that the use of trained dogs leads to earlier detection of infected persons at a lower cost. This helps separate asymptomatic carriers quickly as each dog has a screening capacity of 250 samples per hour, which is effective enough to be used in certain places to control transmission of the disease.

According to the recommendations of the World Health Organization (WHO), the most critical issue in adopting strategies and policies to mitigate the spread of the disease is the ability of screening large number of people, which is either impossible or difficult to do using hitec devices, especially in low-resource countries and settings. So it may be hypothesized that a defined population or environment can be quickly screened at a lower cost by using trained dogs. Therefore, this study aims to evaluate the feasibility of training dogs for detection of COVID-19 cases.

## Methods

### Study design

This study was based on a hypothesis that assumes the sniffer dogs are able to detect metabolic changes in human body, which is caused by the pathological activities of SARS-Cov-2 virus. For this purpose, we designed a training process to condition six male and female dogs from five different breeds including German shepherd, German black, Labrador, Golden retriever, and Border gypsy. The study was conducted into two different training sectors. One study on participants’ pharyngeal secretions samples tested by RT-PCR as the gold standard method to detect positive and negative cases and another study on participants’ face masks and clothes using, who were already tested positive or negative using RT-PCR. Patents were from cases hospitalized in different ICUs across city of Tehran. Negative cases were healthy individuals who were RT-PCR negative for SARS.CoV-2 virus. Finally, the verification test was carried out in a single-blind process.

This study was approved by Iran’s Ministry of Health, Treatment, and Medical Education (IR.AJAUMS.REC.139.055). All dog trainers were fully equipped with proper Personal Protection Equipment (PPE) when working with dogs and the samples.

### Sniffer dogs’ details

Study dogs belonged to the SK9 Dogs Training School. Each dog’s training background, age, and gender had to be taken into account. Since there was no evidence on the success rate of species for detecting the COVID-19 virus, our study dogs were selected from different species and ages. The dogs’ characteristics are shown in Table 1.

**Table 1:** Dogs’ characteristics

Name	Age (year)	Gender	Species
Lexi	1	Female	Labrador
Sami	2	Male	Border gypsy
Saray (SY)	2	Female	German shepherd
Kuzhi (KZH)	1.5	Female	German black
Marco (MRC)	1.5	Male	Labrador
Zhico	3	Male	Golden retriever

### Dog training

Three dogs including one German shepherd, one Labrador, and one Border gypsy were intensively trained by the classical conditioning method for 7 weeks [30–32]. They were introduced to the pharyngeal secretions of both and COVID-19 patients and healthy individuals. The specimens were daily transported from the hospital laboratories to the training site under safe and standard conditions. These samples were placed in groups of 10 consisting of one to three positive samples for COVID-19 inside a dogs training wheel. Positive samples were obtained from the patients admitted to the ICU and were taken from both male and female patients at different ages before and after taking medicines. It should be noted that some patients were completely healthy before contracting COVID-19, while some had underlying diseases such as diabetes, coronary diseases, heart failure, renal failure, respiratory disease such as asthma and Chronic Obstructive Pulmonary Disease (COPD), and Senecavirus A (SVA). In the second section of the study, the training set included face masks and clothes and another three dog were trained using 1300 clothes and 1300 face masks of Covid-19 patients. These stuffs had been worn for 24 h before being used as samples. The control samples for this section included the same hospital clothes and masks from the patients who were admitted in the hospital but were proven by RT-PCR to be not COVID-19 case.

### Safety and protection protocols

#### Testing

Before startingproject, all training team members as well as dogs were tested for COVID-19 using RT-PCR method to make sure that they were not themselves infected by the virus. In the course of the study also, the training team members were tested frequently on days 1, 21, 35, and 49. Two weeks after the final verifications, all team members were tested again to make sure they were infected during the past week. Also all protection measures were taken to protect the dogs. Dogs were quarantined for 2 weeks after the testing events. They underwent RT-PCR tests 2 weeks before and after the testing event and all were negative for SAR-CoV-2 contamination.

#### Personal protection

All team members were highly required to use th PPE, face masks, and shields similar to those used in ICUs.

#### Quarantine

All training team members stayed 24 h/7 days in the training site during the whole study period of time.

#### Samples delivery

Pharyngeal secretion samples, face masks, and clothes were safely delivered to the site in sealed boxes.

#### Sanitizing

Training site, training equipment and the dormitory of the team were sanitized twice a day during the training period.

#### Dogs condition

The dogs were kept in standard cages under standard conditions and were fed with high-quality dog foods. It should be noted that no forceful training equipment were utilized.

### Verification test

In late April 2020, following 7 weeks of intensive training where each dog underwent the training process for averagely about 1000 times, and nearly 120 tests were performed during the course, the verifying tests were conducted for both groups of dogs. For the verification test of the first group of dogs, 80 samples of pharyngeal secretions that 26 and 54 were positive and negative respectively (Negative samples were from healthy individuals) were used in a single-blind fashion. Fr the second group of the dogs 120 samples consisting of clothes and face masks were used that of them 50 and 70 were positive and negative respectively. Negative cases were identified by PCR. However, the negative symptoms of the subjects were checked 2 weeks before and after the test. Notable to mention that the samples were taken from different medical centers and people with various levels of disease severity. The samples were used from the population of Tehran in Iran, and considering that Tehran is a homogeneous sample of the whole country and to be considered socio-economic; positive samples were collected from several different hospitals for maximum diversity.

At the end of the study, none of the dogs had COVID-19 and were completely healthy.

### Statistical analysis

Epidemiological and statistical tests including sensitivity or True Positive Rate (TPR), specificity or True Negative Rate (TNR), positive condition, negative condition, False Negative Rate (FNR), False Positive Rate (FPR), Positive and Negative Likelihood Ratios (LR+/LR-) were performed and the data were analyzed using the SPSS software, version 23.

## Result

In sector 1 of the study, after completing training process, dogs were introduced to the throat and nasopharengeal samples. Dogs were able to identify positive samples among all type of samples with an accuracy of over 80%. In the verification test, 80 samples in test tubes were used in eight sets of ten (both positive and negative) includig 26 positive samples that dogs identified 17 correctly, and 54 negative samples that surprisingly dogs identified 48 of them correctly. Nevertheless, the performances of the three trained dogs were slightly different. The German shepherd Saray (SY) and), the German black dog Kuzhi (KZH) successfully identified six out of the ten positive samples in a set of 30 positive and negative samples. The third dog in this experiment was Marco (MRC), a Labrador that identified five positive samples out of a set of 20 that of them 6 and 14 were positive and negative respectively (Table 2).

**Table 2:** Performances of different dogs

	Positive condition		Negative condition		Predicted positive condition	Positive condition	Predicted negative condition	Negative condition
	True positive	False negative	False positive	True negative				
SY	6	4	3	17	9	10	21	20
KZH	6	4	2	18	8	10	22	20
MRC	5	1	1	13	6	6	14	14
Total	17	9	6	48	23	26	57	54

**Fig. 1:**
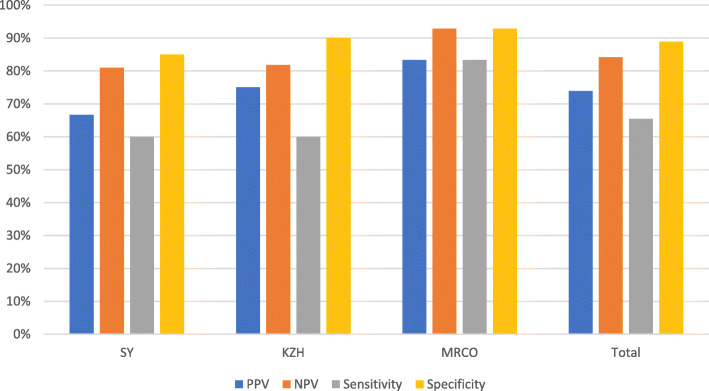
Statistical results of the dogs’ performances

In average, three dogs detected 17 out of the 26 positive samples making a sensitivity of 65%. Moreover, averagely the correct detection rate of the negative samples was 89% for three dogs (Table 3) (Fig. 1).

**Table 3:** Statistical results of the dogs’ performance

	Sensitivity or true positive rate (TPR) %	False negative rate (FNR) %	False positive rate (FPR)	Specificity or true negative rate (TNR)	Positive likelihood ratio	Negative likelihood ratio
**SY**	60%	40%	15%	85%	4	0.4706
**KZH**	60%	40%	10%	90%	6	0.4444
**MRC**	83%	17%	7%	93%	11.667	0.1795
**Total**	65%	35%	11%	89%	5.8846	0.3894

In the sector 2 of the study**,** the testing set included 50 positive (clothes and masks from RT-PCR positive patients) and 70 control (from RT-PCR negative people) samples. Out of the 50 positive samples, 43 were correctly identified by Lexi, Sami and Zhico. Moreover, out of the 70 negative samples, 65 were correctly detected by them. The sensitivity of this experiment was as high as 86% with a specificity of 92.9%. Additionally, the positive and negative predictive values were found to be 89.6 and 90.3%, respectively (Table 4).

**Table 4:** The overall performance of three dogs in the sector 2of the study

	Condition Positive		Condition Negative											
	True positive	False negative	False positive	True negative	Predicted positive condition	Positive condition	Predicted negative condition	Negative condition	Sensitivity or true positive rate (TPR) %	False negative rate (FNR) %	False positive rate (FPR)	Specificity or true negative rate (TNR)	Positive likelihood ratio	Negative likelihood ratio
**Face Masks and clothes**	43	7	5	65	48	50	72	70	86.0%	14.0%	7.1%	92.9%	12.04	0.15077

## Discussion

First of all it must be mentioned that due to the observance of appropriate protection protocols, neither the dogs nor the trainers were infected with COVID-19 during 10 weeks of training, which was a great achievement.

Since the historical case report by Williams et al. in 1989 [17], there have been few experiments in the utility of the canine olfactory system for medical purposes. These experiments were mostly related to the detection of ovarian, prostate, colorectal, and lung cancer [20, 22, 23, 26, 27, 33, 34]. However, the use of canine olfactory system in other areas such as infectious diseases has been rarely studied. The current study aimed to investigate the applicability of canine olfactory system in detecting COVID-19 cases and carriers of SARS-C0v_2. In this regard, a recent pre-print paper showed the efficacy of dogs in differentiating the COVID-19 positive and negative cases using armpit sweat [35]. There was a study by Jendry et al. that started after ours. Their aim was to detect Covid-19 samples using dogs and They showed that dogs were able to correctly detect 94% of positive and negative samples. Their sensitivity and specificity were 82.63 and 96.35% respectively [36]. They used larger number of samples in verification test, howere all RT-PCR tests were performed by themselves. In our study, the RT-PCR tests were run by hospital laboratories to avoid the possible bias.

Regarding the results we have achieved, it can be declared that his research has successfully proven a the concept of the presence of a specific and traceable odour in people with COVID-19 disease. In the sector 1 of the study, the dogs successfully distinguished over 65% of the positive specimens and 89% of the negative samples and inthe sector 2 where clothes and masks of participants were used, dogs could correctly detect 86% of the positive and 92.9% of the negative samples. It should be noted that the samples were taken from people with different clinical conditions on different days and from different hospitals to minimise the possible environmental effects. It can be certainly claimed that the presence of SARS-CoV-2 virus in human body leads to production and release of a specific odour, which does not exist innegative sample. We assume that a specific chain of chemical and metabolic reactions causes that specific odour in affected people with Covid-19.

In the sector 1 of the study, the obtained sensitivity and specificity were 65 and 89% respectively, however in the sector 2, these values were as high as 86 and 92.9%, respectively, which are somehow comparable to those of laboratory diagnostic kits such as RT-PCR [37, 38]. As we currently know, there are at least 3481 VOCs in human breath, so we hope by using them it can be possibe to design biosensors and electronic noses to detect Covid-19. Currently, it is already proven that the VOCs are associated with several malignancies. For example, 4-methyl decane, dodecane, and undecane have been reported to be associated with malignant melanoma [39].

In terms of diagnosis speed, dogs are real-time detectors that are highly required in the setting of pandemics. Furthermore, dogs are supposed to have better performance with more intense training. Thus, such detecting dogs can be used to identify suspicious COVID-19 cases in places with a high population density, such as airports, passenger terminals, and important national centers.

We suggest there were some limitations with our study. Firstly we did not have a prevalence rate. Secondly it was not possible for us to double check the people who were RT-PCR negative (our controls) by a second RT-PCR test. Thirdly, at the nearly end of the dog training phase, Iran managed to substantially cut the number of new cases, so we were unable to raise the number of samples in regard of achieving statistically stronger results. Lastly if we could reduce the false negatives rate to a figure nearly zero, then the sensitivity would largely increase.

## Conclusion

Dogs can be trained to identify people with COVID-19 disease and can be used as a reliable tool in limited screening programs. This study was a limited experience and could serve as a basis for other researchers to inspire. Identification of the VOCs associated with COVID-19,provides a great opportunity todevelop biosensors for screening of mass population.

## Data Availability

The datasets used and/or analysed during the current study are available at Shiri, Mahdi, Ahmadi Marzaleh, Milad.

## Abbreviations

CT-scan: Computed Tomography Scan
PCR: Polymerase Chain Reaction
VOCs: Volatile Organic Compounds
WHO: World Health Organization
PPE: Personal Protection Equipment
COPD: Chronic Obstructive Pulmonary Disease
SVA: Senecavirus A
TPR: True Positive Rate
TNR: True Negative Rate
FNR: False Negative Rate
FPR: False Positive Rate
PPV: Positive Predictive Value
NPV: Negative Predictive Value
